# Continuous Bamboo Fibers/Fire-Retardant Polyamide 11: Dynamic Mechanical Behavior of the Biobased Composite

**DOI:** 10.3390/polym14020299

**Published:** 2022-01-12

**Authors:** Louise Lods, Tutea Richmond, Jany Dandurand, Eric Dantras, Colette Lacabanne, Jean-Michel Durand, Edouard Sherwood, Gilles Hochstetter, Philippe Ponteins

**Affiliations:** 1 CIRIMAT, Université de Toulouse, PhyPol, 118 Route de Narbonne, 31062 Toulouse, France; louise.lods@univ-tlse3.fr (L.L.); tutea.richmond@univ-tlse3.fr (T.R.); jany.lods@univ-tlse3.fr (J.D.); eric.dantras@univ-tlse3.fr (E.D.); 2 Cobratex, 1 Allée Orchidée, Local 8—ZA Activestre, 31390 Carbonne, France; jean-michel.durand@cobratex.com (J.-M.D.); edouard.sherwood@cobratex.com (E.S.); 3 Arkema, 420 Rue d’Estienne d’Orves, 92700 Colombes, France; gilles.hochstetter@arkema.com; 4 Expleo Toulouse, 13 Rue Marie Louise Dissard, 31300 Toulouse, France; philippe.ponteins@expleogroup.com

**Keywords:** technical bamboo fibers, bamboo fibers/thermoplastic composites, fire retardant, thermogravimetric analysis (TGA), dynamic mechanical analysis (DMA), glassy/rubbery modulus

## Abstract

A biobased composite was generated from bamboo fibers (BF) and a polyamide 11 (PA11) matrix. In order to fulfill security requirements, a PA11 already containing a flame retardant (FR) was chosen: This matrix is referred as PA11-FR. In this work, the effects of flame retardant (melamine cyanurate) on the composite properties were considered. In the calorimetric study, the glass transition and melting temperatures of PA11-FR were the same as those of PA11. The melamine cyanurate (MC) had no influence on these parameters. Thermogravimetric analysis revealed that PA11-FR was less stable than PA11. The presence of MC facilitated thermal decomposition regardless of the analysis atmosphere used. It is important to note that the presence of FR did not influence processing conditions (especially the viscosity parameter) for the biosourced composite. Continuous BF-reinforced PA 11-FR composites, single ply, with 60% of fibers were processed and analyzed using dynamic mechanical analysis. In shear mode, comparative data recorded for BF/PA11-FR composite and the PA11-FR matrix demonstrated that the shear glassy modulus was significantly improved: multiplied by a factor of 1.6 due to the presence of fibers. This result reflected hydrogen bonding between reinforcing fibers and the matrix, resulting in a significant transfer of stress. In tensile mode, the conservative modulus of BF/PA11-FR reached E’ = 8.91 GPa. Upon BF introduction, the matrix tensile modulus was multiplied by 5.7. It can be compared with values of a single bamboo fiber recorded under the same experimental conditions: 31.58 GPa. The difference is partly explained by the elementary fibers’ lack of alignment in the composite.

## 1. Introduction

Over the last 15 years, interest concerning technological development and processing of biocomposites has tremendously increased [1,2,3,4,5,6,7]. The subject of a thermoplastic matrix reinforced with natural fibres has been widely discussed [8,9,10,11,12,13].

These environmentally friendly composites have many remarkable properties in comparison with conventional composites using glass or mineral fibres due to their low density and low cost [11]. Among all lignocellulosic fibres, bamboo seems to be one of the most useful. Bamboo is abundant on all the continents [14,15], and its quick growth leads to a continuous supply, which is an important advantage for industrial applications [16,17].

The major technological barrier for the use of plant fibres as composite reinforcement is their thermal stability. Indeed, the processing temperature for the composites and, therefore, the range of use for these materials is contrained. Thermoset matrices and more particularly matrices such as polyester [18,19,20] or epoxy [21,22,23,24,25,26,27,28] due to their low processing temperature are compatible with these requirements. Thermoplastics such as polylactic acid (PLA) [29,30,31], polypropylene (PP) [32], poly(vinyl chloride) (PVC), and polyamides (PA) [33,34] may also be used as matrix. For the sake of recyclability, the choice of a thermoplastic matrix is highly relevant.

In addition, to produce composites reinforced with natural fibres, it is necessary to consider several technological issues for the choice of the polymer. Bamboo fibres degrade above 200 °C [35,36]: Accordingly, the polymeric matrix must be processed below this temperature. Moreover, the necessity to assure interface compatibility leads to the choice of a polar matrix [37,38]. In order to reduce the environmental impact of the final composite, biosourced polymers may be used as a matrix (>51% from plant resources).

To meet these criteria, polyamides (PA) were selected as a suitable matrix material. The most well-known polyamides, PA6,6 and PA6, are incompatible with the thermal stability of natural fibres due to relatively high processing temperatures. However, as the length of aliphatic blocks for polyamides increases, the melting temperature decreases and the processing temperature becomes compatible with the use of lignocellulosic fibres. Consequently, PA11 [39,40] was selected as a matrix for the generation of bamboo-reinforced composites. The polymer, PA11, is produced from a pyrolysis product of castor oil, a renewable resource. For the targeted industrial applications, the polymer chosen must also meet the standards for fire, smoke, and toxicity. Therefore, a polyamide containing fire-retardant (FR) additives to improve the fire resistance was obtained from Arkema. This polymer will be referred to as PA11-FR. Fire-retardant additives may modify the thermal decomposition of polymers in order to limit the generation of combustible products. Others may promote the formation of a char layer at the surface of the degrading polymer to inhibit heat feedback from the combustion zone. Still others liberate species capable of disrupting radical combustion reactions to the gas phase [41,42,43,44,45,46].

To characterize the used FR, the PA11-FR was examined using infrared spectroscopy. The thermal behaviour of the polymer was etablished using differential scanning calorimetry (DSC) and thermogravimetric analysis

This new matrix can be implemented for composite formation using methods previously described [36]. Mechanical properties were evaluated using DMA in order to determine the complex modulus of matrices and composites in shear and tensile modes.

## 2. Materials and Methods

### 2.1. Materials

#### 2.1.1. Continuous Bamboo Fibers

Phyllostachys viridiglaucescens culms (traded by Cobratex/France) were harvested in the Southwest of France with a yearly average precipitation of 800 mm, a relative moisture of 80%, and a median annual temperature of 16 °C. All the bamboo canes studied were at least 3 years old. Indeed, research shows that the chemical composition of fibres and their lignin content continues to evolve throughout the first 3 years of development; however, this does not seem to affect mechanical properties [47].

Phyllostachys viridiglaucescens is a species of monocotyledonous plants of the Poaceae family, subfamily of Bambusoideae. They are giant bamboos, tracing 4 to 7 m/year, with stems (culms) reaching 8 to 13 m high and 5 to 10 cm in diameter. The preferred location for Phyllostachys bamboo is a warm temperate climate, although this species is particularly resistant to cold, which allows it to be grown in Europe [48].

What is called fibres in this article is actually technical fibres; these were extracted from bamboo sticks by an innovative hybrid process, mixing chemical and mechanical approaches (the extraction did not require any high temperature or steam explosion). Bamboo sticks are immersed in water to cause them to swell until submersion and then they are laminated. The bamboo is immersed in a 1% alkaline solution for 2 h at 70 °C under agitation. After this step, it is washed with distilled water to neutralize the pH and remove residual elements before being rolled again. Finally, the fibres are extracted by brooming. The average diameter of these fibres is about 400 microns and the yield of this extraction process is about 50%. The internode length conditions the limit size of extracted fibres. For a 3-year-old culm, the internode dimension is about 25–30 cm. The fibres were approximately 25 cm long with a cross-section about 100 μm.

**Figure 1:**
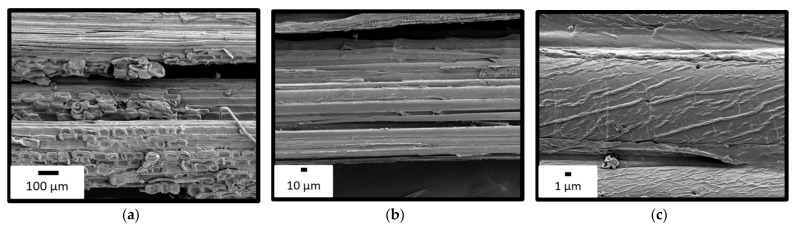
SEM images of fibers extracted in the laboratory at different magnifications: (**a**) Wick of technical fibers; (**b**) Technical fiber (diameter ≈ 400 µm); (**c**) Elementary fiber (diameter ≈ 20 µm).

Representative fibres extracted by this process in the laboratory were observed by SEM (Figure 1). The microscopy showed a technical fibre and its constituent elementary fibres. Some parenchyma (plant matrix) cells are still visible at the bottom of Figure 1 a. However, the fibres appeared to be quite bare, which is favourable for a good interface with the polymer matrix. Moreover, the fibres were not damaged by the treatment and, therefore, their mechanical properties were maintained. We designated as bamboo fibres (BF) the wicks of the technical fibres constituted of elementary fibres. This hierarchy is analogous with the one of glass or carbon fibres.

#### 2.1.2. Biobased Polyamide Matrix

PA 11 is a biosourced thermoplastic synthesised by Arkema (France) under the registred trademark Rilsan PA11 D30 NAT. It is made from a renewable source, castor oil, and contains a high amount of renewable carbon, according to ASTM D 6866 (calculated) more than 90%. Its water absorption at ambient temperature is less than1%. As a result of its melting temperature of 190 °C, it is well adapted to constitute the matrix of natural fibre-reinforced composites. It was supplied in granular form for the composites’ processing.

PA11-FR is a biobased flame-retardant polyamide synthesised by Arkema as Rilsan^®^ MB 3000 NAT (trade name). FR is a halogen-free flame retardant.

Melamine cyanurate (MC) is an ecologically aware flame retardant, formulated in the early 1980s in Japan. This nitrogen-containing fire retardant is attracting more and more attention because of its numerous advantages, for example, non-toxicity, low loading level, and good compatibility with thermoplastics. Melamine cyanurate is especially valuable for enhancing the fire safety of nitrogen-based polymers and particularly polyamide. It can also be employed in thermosets and in numerous matrices [43]. At room temperature, the water uptake of PA11-FR for a 50% humidity rate is 0.6 ± 0.2%. By reason of its low melting point at 189 °C, it is well matched for constituting the matrix of natural fibres’ composites. It was supplied in granular and film forms.

#### 2.1.3. Processing of Composites

Polyamide films were cut into rectangles of dimensions of 10 × 1 cm. Fibres were then laid in a unidirectional layer between two polyamide films. To reach a reinforcement rate of 60% in mass, the calculated bamboo fibres/polyamide ratio was 3:2. The system was then pressed and heated at 198 °C for 15 min. After all of this, samples were naturally cooled and stored at ambient temperature for about 20 h before testing. To make the reference matrix samples, films were stacked to be as thick as composites, and then pressed at 198 °C.

Samples were designated as BF/PA11 and BF/PA11-FR for composites with PA11 matrix and PA11-FR matrix, respectively.

### 2.2. Methods

#### 2.2.1. Scanning Electron Microscopy

Bamboo fibres and composites were studied by Scanning Electron Microscopy (SEM). The observation was performed with a JEOL JSM 7800F Prime (Tokyo, Japan). This device is a high-end field emission scanning electron microscope, which allows reaching sub-nanometric resolution at all acceleration voltages but also has high analytical capacities (up to 600 nA of current). It is equipped with an EDS SDD spectrometer.

The applied acceleration voltage was 3 to 5 kV. The composites were cryofractured in liquid nitrogen before observation with the purpose of studying the fibre/matrix interface. All samples were metallized with platinum to allow the flow of charges and to avoid their accumulation on the surface.

#### 2.2.2. Fourier Transform Infrared Spectroscopy

Fourier transform infrared spectroscopy/attenuated total reflectance (FTIR/ATR) spectra of matrices were recorded employing a Nicolet 5700 FTIR instrument (Thermo Fisher Scientific, Waltham, MA, USA) equipped with a reflectance accessory consisting of a KBr beam splitter and an MCT/B detector. The ATR tool is a Smart Orbit with a type IIA diamond crystal (refractive index 2.4). Samples were directly placed on the crystal and softly squeezed with a Teflon point to guarantee good contact. For each sample, 32 interferograms were accumulated in the 4000–450 cm^−1^ region, co-added to produce an average signal with a nominal resolution of 1 cm^−1^ using Omnic 8.0 (Thermo Fisher Scientific). The background signal was acquired from the clean diamond crystal before every experiment; this signal was deducted from the sample spectra.

#### 2.2.3. Differential Scanning Calorimetry

DSC analyses were done using a DSC Pyris calorimeter (PERKIN ELMER, Waltham, MA, USA) with an unfilled pan as reference. The apparatus has a temperature precision of ±0.1 °C and an enthalpy precision of ±2.10^−4^ J kg^−1^. Samples were placed in closed aluminum pans with a mass fluctuating between 5 to 10 mg. Measurements involved three heat-up runs and two cool-down cycles. Experiments were conducted between 0 to 250 °C at a speed rate of 10 °C min^−1^ under a nitrogen flow. Glass transition temperature (Tg) and heat capacity (ΔCp) were obtained by the tangent method while the melting temperature ™ was determined at the peak top.

#### 2.2.4. Thermogravimetric Analysis

Thermogravimetric analysis (TGA) was carried out on a TGA Q50 (TA Instruments, New Castle, DE, USA) with a 0.1-µg mass sensitivity and a 0.1-°C temperature imprecision. Mass evolution was measured for a linear increase of temperature under nitrogen (inert atmosphere) or air (oxydant atmosphere) with an initial sample amount of 5–10 mg. Dynamic tests were performed from room temperature to 1000 °C at a 10 °C min^−1^ temperature speed rate.

#### 2.2.5. Dynamic Mechanical Analysis

Mechanical properties were investigated by Dynamic Mechanical Analysis (DMA) with an Advanced Rheometric Expansion System setup (Rheometric Scientific, New Castle, DE, USA). Experiments were conducted under dynamic strain, on parallele-piped samples, in the temperature range from –130 °C to 150 °C, with a heating rate of 3 °C min^−1^, at an angular frequency of 1 rad s^−1^, under nitrogen flow. As soon as the first scan was finished, samples were cooled down at 3 °C min^−1^ and the second scan was carried out using the previous parameters.

In the isofrequency mode, the temperature variation of the complex modulus M* is given by the following relationship:

$$M_{\omega 0}^{*}\left(T\right)=M_{\omega 0}^{\prime }\left(T\right)+i.M_{\omega 0}^{\prime \prime }\left(T\right)$$

where $M_{\omega 0}^{\prime }\left(T\right)$ is the conservative modulus and $M_{\omega 0}^{\prime \prime }\left(T\right)$ is the dissipative modulus.

Under applied strain, the Maxwell model describes the dynamic mechanical behaviour. For the isofrequency mode, it is given by the equations below:

$$M_{\omega 0}^{\prime }\left(T\right)=Mr+\left(Mg-Mr\right)\frac{\omega_{0}^{2}\tau \left(T\right)2}{1+\omega_{0}^{2}\tau \left(T\right)2}\;M_{\omega 0}^{\prime \prime }\left(T\right)=\left(Mg-Mr\right)\frac{\omega_{0}\tau \left(T\right)}{1+\omega_{0}^{2}\tau \left(T\right)2}$$

where Mr is the rubbery modulus, Mg is the glassy modulus, $\omega_{0}$ is the frequency of strain oscillation, and τ is the relaxation time. Shear conservative and dissipative moduli are designated as $G_{\omega 0}^{\prime }$ and $G_{\omega 0}^{\prime \prime }$, tensile conservative and dissipative moduli are defined as $E_{\omega 0}^{\prime }$ and $E_{\omega 0}^{\prime \prime }$.

Torsion measurements were carried out using a dynamic shear strain of 0.1%, and elongation measurements were done in the linear zone under a dynamic tensile strain of 0.15%.

## 3. Results and Discussion

### 3.1. FTIR

**Figure 2:**
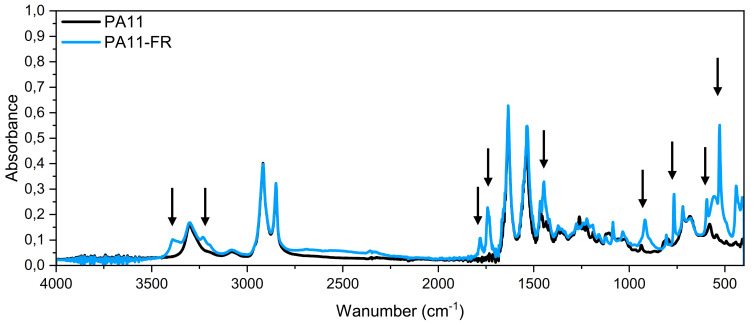
FT-IR spectra of PA11 and PA11-FR.

FT-IR spectra of PA11 and PA11-FR are shown in Figure 2. Classical absorption bands of polyamide were present in these spectra but some differences between the two matrices were observed, pointed out by arrows. These peaks and the vibration band assignment characteristics of the PA11-FR fire retardant are listed in the Table 1.

In the literature, these bands have been assigned to the internal vibrations of melamine cyanurate molecules.

According to Sangeetha et al. [49], “melamine cyanurate complex is held together by an extensive two-dimensional network of hydrogen bonds between the two compounds”. Peaks, put into evidence in Figure 2, stemmed from lattice and internal vibrations of melaminium cations, cyanuric acid anions, H_2_O molecules, and melaminium residues, which created hydrogen bonds.

The band at 3390 cm^−1^ corresponded to the NH_2_ symmetric stretching type of vibrations of triazine groups. It was a blue shift of a band that usually occurs in melamine crystal. The peak at 3232 cm^−1^ was assigned to the formation of hydrogen bonds between NH_2_/NH groups. The stretching vibration of the carbonyl groups C=O at 1780 cm^−1^ was attributed to cyanate anions. The peak at 1741 cm^−1^ was assigned to NH_2_ scissoring. The benzene ring had two absorption peaks assigned to the vibrations of C-N and C=N bonds. These appeared in the zone of 1450 cm^−1^ to 1500 cm^−1^ and 1530–1600 cm^−1,^ correspondingly, which suggested the presence of cyanuric acid. A similar band was shifted to lower frequency, at 1447 cm^−1,^ in our spectra. The bands at 808 cm^−1^ and 766 cm^−1^ corresponded to ring-sextant, out-of-plane bending type of vibration, and the band at 527 cm^−1^ was attributed to the side chain in plane C-N bending vibration.

### 3.2. Thermal Transitions

**Figure 3:**
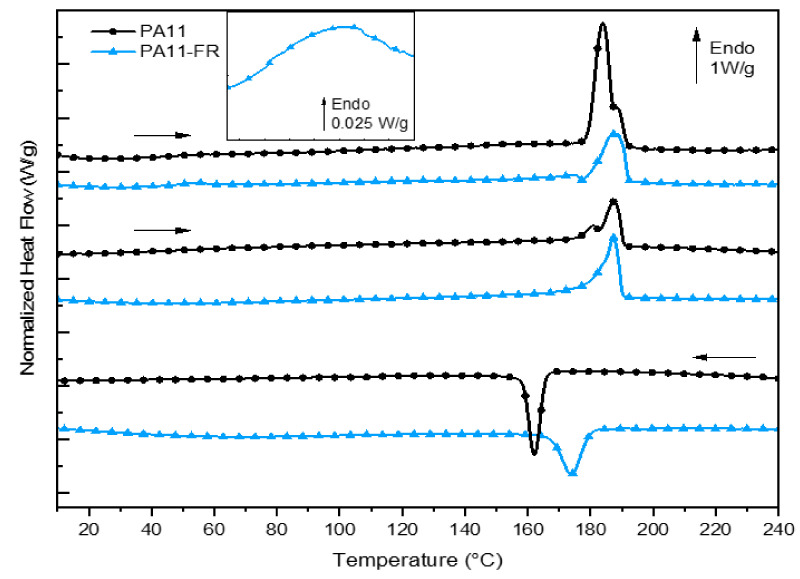
DSC curves of PA and PA11-FR and zoom-in the PA11-FR glass transition.

Figure 3 shows the calorimetric responses, i.e., heat flux normalized to mass, for each of the two matrices: PA11 and PA11-FR. Thermal parameters and transitions of both polyamides are indicated in Table 2. The Tg/Tm ratio (with temperature in Kelvin) was determined and was about 0.7. This calculation is coherent with the Van Krevelen relation, valid for a large number of unsymmetrical polymers [50].

#### 3.2.1. PA11

In the first heat-up ramp, presented in Figure 3, the glass transition temperature of PA 11 was evidenced at 46 °C. The melting of crystallites began at around 172 °C and ended at 193 °C. The presence of a double melting peak for this matrix was already demonstrated in previous work [39]. It is explicated by the coexistence of crystallites of diverse morphology: crystallites of different shapes and/or size distribution of the crystallites. The degree of crystallinity of the samples is determined from the below relationship:

$$\chi_{c}\;(\%)=\frac{\Delta \mathrm{Hm}}{\Delta H\infty }\;\times \;100$$

The ΔHm is the melting enthalpy of polyamide 11 inferred from the area under the melting curve and ΔH∞ is the theoretical melting enthalpy calculated for 100% crystalline polyamide 11, which is equal to 244 J g^−1^. The degree of crystallinity of PA11 thus calculated was about 37% for the first rise and 15% for the second rise.

#### 3.2.2. PA 11-FR

The glass transition resulted in a jump in heat capacity, which was difficult to demonstrate for aliphatic polyamides. However, for the PA 11-FR matrix, physical aging made it possible to reveal this variation in heat capacity with an overshoot (see zoom-in between 45 °C and 60 °C). It corresponded to the creation of physical bonds between the chain segments of the amorphous phase and occurred in the temperature range (Tg) –50 °C. The ambient temperature was enough to create this new local order. The kinetic of this phenomenon was slow and the physical bonds did not have time to reform between the first and the second sweeps. Consequently, the glass transition temperature was recorded on the first scan and estimated at 42 °C for the PA11-FR matrix. In the heat-up ramp, the endothermic event at the glass transition was not visible: This behavior corroborated that the initial event was corresponding to physical aging. The melting temperature was not modified.

The PA11-FR degree of crystallinity calculated was about 22% for the first scan and 24% for the second rise (taking ΔH∞ = 244 J·g^−1^).

On the first scan, an exothermic event was visible just before the melting peak. According to Descamps and Willart [51,52], that exotherm corresponded to the growth of nucleated crystals at much lower temperatures.

It is worth bearing in mind that the crystallization temperature of PA11-FR is 10 degrees higher than the PA11 one. It may be due to the presence of melamine cyanurate (MC) in PA11-FR. MC is an organic crystalline complex. This compound was used in the form of a powder with an average particle size of a few microns. These small particles could be at the origin of this “faster” crystallization by creating nuclei that initiate crystal growth.

These melting temperature values and crystallinity rates make it possible for the PA11-FR matrix to use the protocol previously established for PA11 in the laboratory [36,39].

### 3.3. Thermogravimetric Analysis

**Figure 4:**
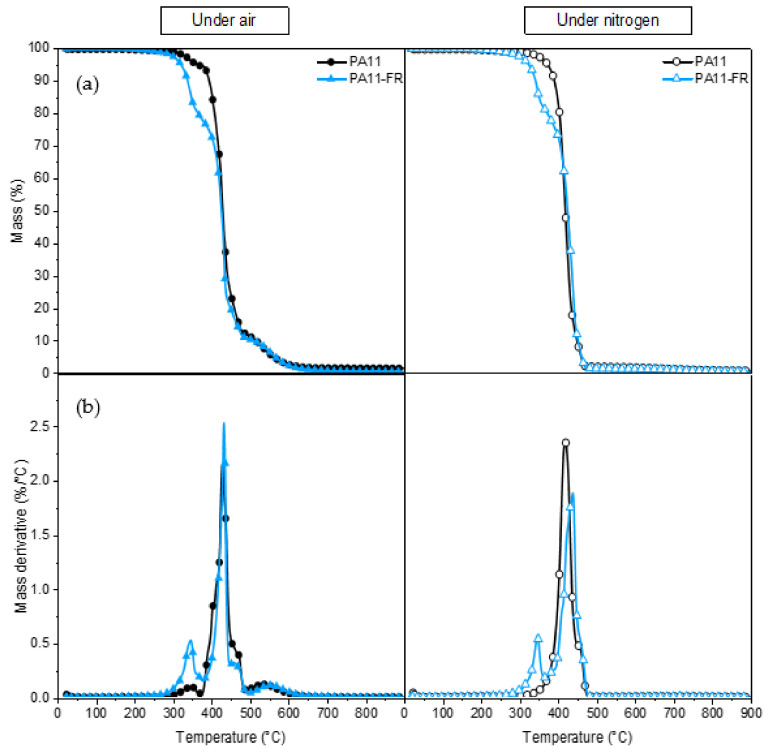
(**a**) TG and (**b**) DTG curves of PA11 and PA11-FR under air atmosphere and nitrogen atmosphere.

Before the processing of composites, the thermal stability of PA11 and PA11-FR was determined by thermogravimetric analysis. Mass loss (TGA) and its derivative (DTG) are traced in Figure 4. Degradation and oxidation can be distinguished by performing a TGA under inert and oxidant atmosphere. Indeed, under nitrogen, only the degradation is highlighted since the oxidation is visible only under air.

#### 3.3.1. PA11

The PA 11 mass evolution and its derivative are shown as a function of temperature, under air (oxidizing environment), and under nitrogen (inert atmosphere). At around 100 °C, a slight loss of mass was observed: It was associated with the desorption of water. PA 11 is not hydrophilic: The amount of water absorbed is of the order of 1%. Under nitrogen, PA 11 is totally degraded between 300 °C and 480 °C: The peak maximum is at 418 °C. Previous studies [53] associated this loss of mass with saturated and unsaturated nitriles, lactams, and hydrocarbons. In air, the first degradation occurred at a lower temperature, i.e., between 300 °C and 375 °C. The degradation under air was maximum at 427 °C, i.e., around 10 °C higher than under nitrogen. A third degradation was also present between 500 °C and 600 °C. This difference in behavior depending on the environment was due to the thermo-oxidation of polyamide.

#### 3.3.2. PA11-FR

Under nitrogen, the degradation underwent two different steps. These two stages of degradation were viewable via two peaks in DTG: The first one was between 300–350 °C and the second one (the maximal degradation) was in the range of 350–480 °C. The second peak was characteristic of PA11 degradation. The first peak corresponded to the melamine cyanurate fire retardant: Melamine cyanurate and its salts are very efficient fire retardants, well known to give good results especially with Nylon 6. Melamine facilitates the thermal decomposition, perhaps because it interferes with the hydrogen-bonding network of the polymer and exhibits basic catalysis. Melamine salts give off acids during thermal decomposition, which also catalyzes the degradation. The evolution of oligomeric fragments at the expense of the evolution of highly combustible caprolactam is one of the main influences of melamine and its salts. Melamine and melamine cyanurate also intensify side reactions of polyamide decomposition, for example, the dehydration of primary amide chain ends, or they cause scission of alkyl-amide –CH_2_-C(O)- bonds and formation of carbodiimide. Other studies by Casu et al. [54] also found that volatilization with fire retardant (with melamine cyanurate) starts at a temperature inferior to the one awaited on the basis of the behavior of the polymer and melamine cyanurate heated distinctly. They suggested a condensed phase, fire-retardant action for melamine cyanurate. In fact, analysis of degradation of polyamide with melamine did not show significant differences compared to the pristine polymer. The traditional method of synthesizing MC in a dispersant, i.e., hot water with alkali salt or NaOH as catalyst, faces many problems. The flame retardancy of the final product can be very weakened as a result of the introduction of the alkali-based catalyst. However, the evolution of ammonia from the polymer was suppressed in the presence of melamine.

### 3.4. Dynamic Mechanical Properties of Composites

Thermal behavior of the PA11-FR matrix showed that it can be implemented according to the protocol established during our previous work [36]. The purpose was now to analyze BF/PA11-FR composites by dynamic mechanical analysis.

#### 3.4.1. Shear Mode

**Figure 5:**
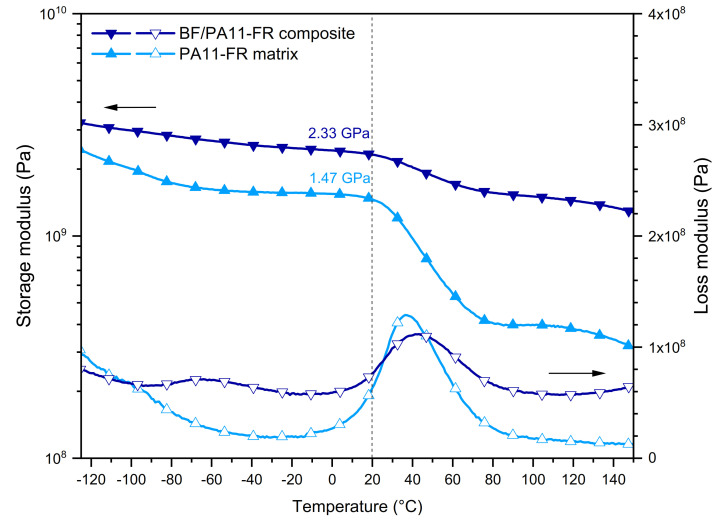
DMA curves in shear mode of PA11-FR matrix and BF/PA11-FR composite.

The shear behavior of 60/40 BF/polyamide single-ply composites was compared with the one of the PA11-FR matrix (Figure 5). The second scan of dynamic mechanical analysis is here presented to disregard the thermal history characteristic to the composites’ processing. The conservative modulus G’ is represented in filled symbols and the dissipative modulus G” by open symbols. Thermograms show the PA11-FR matrix in light blue and composite 60/40 in dark blue.

##### Shear Storage Modulus

On the thermogram of the conservative modulus G’, an important event was visible around 40 °C: It was the viscoelastic transition of the polymer. With bamboo fibers’ incorporation, an improvement of the conservative modulus G’ was discerned for both states: glassy and rubbery. This improvement of modulus in the glassy state highlighted the continuity of matter between the bamboo fibers and the polar polyamide matrix. Moreover, there was a stress transfer between the fibers and the matrix by reason of the formation of static hydrogen bonds between the hydrophilic filler and the amide groups of the matrix. At room temperature (T = 20 °C), the matrix modulus was G’m = 1.47 GPa. Note that it was significantly increased regarding the value of the one of polyamide 11 (0.56 GPa) since the FR particles played the role of a rigid filler. For the composite with 60 wt % of fibers, the shear modulus was G’ = 2.33 GPa. The increase of the vitreous shear modulus upon the introduction of fibers indicated the existence of physical static interactions between matrix and fibers.

In the rubbery state, PA11-FR matrix and BF/PA11-FR composite had different behaviors: The first one showed a linear polymer flow above its glass transition while the composite acted as a network with a real rubbery plateau. In composites, bamboo fibers behaved as additional topological nodes. The viscoelastic step corresponded to the change of static hydrogen bonds into dynamic hydrogen bonds. Analogously with the glassy modulus, the rubbery moduli of the PA11-FR matrix and the BF/PA11-FR composite at 80 °C were compared. It was interesting to observe that the influence of the fibers remained of the same order of magnitude. The point to emphasize is the high value of the rubbery modulus of the composite: 1.56 GPa.

##### Shear Loss Modulus

For the BF/PA11-FR composite, the thermogram of the dissipative modulus G” (empty symbols) showed two modes of relaxation β and α, in the order of increasing temperatures.

The β relaxation at −65 °C was due to the local mobility of amide-water groups in the amorphous phase. This mode was observed for the composite but not for the polyamide. This result showed that the presence of bound water was inherent in the bamboo.

The α relaxation of the composite was located at Tα = 42 °C while it was at Tα = 36 °C for the polyamide. The introduction of the fibers was accompanied by physical interactions that stiffened the matrix and limited the dissipative effect: For the composite, the amplitude of the peak was smaller than for the matrix.

#### 3.4.2. Tensile Mode

**Figure 6:**
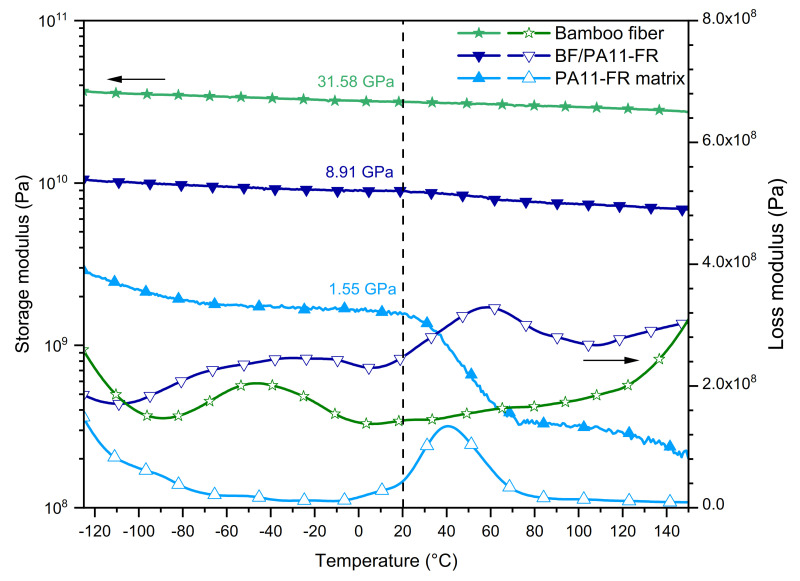
DMA curves in tensile mode of polyamide PA11-FR, bamboo fibers/polyamide PA11-FR composite, and bamboo fiber.

Figure 6 shows the evolution of the E’ tensile storage modulus (filled symbols) and the E’’ loss modulus (open symbols) function of temperature for the polymeric matrix (light blue), the composite (dark blue), and the single bamboo fiber (green), respectively.

##### Tensile Storage Modulus

The elastic part presented an analogous behavior to the shear mode; thus, the introduction of BF improved the modulus in the entire temperature range. At room temperature, the composite storage modulus was E’ = 8.91 GPa for the composite and E’ = 1.55 GPa for the polymer. Consequently, the step between the glassy and the rubbery plateau was less marked for the composite than for the pristine polymer. For the composite, the viscoelastic step was also less important in the tensile mode than in the shear mode. This difference is explained by the fact that, in the tensile mode, the composite was stressed in the direction of fibers so that there was only a minor contribution of the flow of the polymeric matrix. The composite had a quasi-stationary elastic modulus at 9 GPa.

The E’ conservative modulus of the single bamboo fiber exhibited a very large glassy plateau with a modulus at 20 °C of 31.58 GPa. This value is consistent with previous studies carried out in the laboratory [36] and with literature data [55].

##### Tensile Loss Modulus

The α relaxation of BF/PA11-FR was located at 58 °C, i.e., 18 °C above the glass transition of the matrix. This shift may be explained by polar interactions between bamboo fibers and polyamide matrix. On the high-temperature side, the increase of energy loss might have been due to the viscoelasticity of bamboo fibers. This hypothesis is supported by previous data recorded on bamboo strips obtained from the same bamboo species, reported by Richmond et al. [56]: They observed by DMA in the tensile mode a viscoelastic relaxation peak at 200 °C.

The β mode was observed around –50 °C for the composite, while it was missing for the pristine polymer. As previously mentioned for shear data, this mode was due to rotational movement of the amorphous phase amide groups. This hypothesis was confirmed by the observation on the tensile loss modulus of this β mode for the single bamboo fiber (Figure 6). At the lowest temperature, the end of the γ mode was observed for PA11-FR matrix and bamboo fibers. It was not observed for the composite due to the lower contribution of the matrix. Overall, in the composite, the dissipative effect was significant at medium and low frequency, which gave it a good resilience.

## 4. Conclusions

A bio-based, fire-retardant PA 11 was investigated. The infrared spectroscopy showed us the FR is a melamine cyanurate. This additive is well known to form hydrogen bonds. It was, therefore, necessary to check whether these had an influence on the thermal properties of the polymer. The DSC study presented a glass transition temperature at 42 °C and a melting peak at 189 °C. Furthermore, these scans put into evidence a crystallization phenomenon probably due to MC. In TGA, the PA11-FR matrix was less stable than PA11. Melamine cyanurate facilitated the thermal decomposition regardless of the atmosphere used for the analysis. This fire retardant probably interfered with the hydrogen bonding network of the polymer and exhibited basic catalysis. Finally, this thermal study demonstrated that PA11-FR complies with processing conditions.

Continuous bamboo fibres-reinforced PA 11-FR composites, single-ply, with 60 m % of BF were processed and analyzed by dynamic mechanical analysis. In the shear mode, comparative data recorded on BF/PA11-FR composite and PA11-FR matrix highlighted a significant enhancement of the shear glassy modulus, which was multiplied by a factor 1.6 at 20 °C. This result reflected hydrogen bonding between bamboo fibres and polyamide matrix, resulting in a transfer of stress between reinforcing fibres and matrix.

In the tensile mode, the conservative modulus of BF/PA11-FR, at 20 °C, reached E’ = 8.91 GPa. Upon the introduction of bamboo fibres, the tensile modulus of the matrix was multiplied by a factor 5.7. It can be compared with the values of a single bamboo fibre recorded in the same experimental conditions: 31.58 GPa. The difference is partly explained by the lack of alignment of elementary fibres in the composite.

## Figures and Tables

**Table 1:** Vibration bands’ assignment of the fire retardant.

Experimental Position (cm^−1^)	Assignment
3390	NH_2_ symmetric stretching type of vibrations of 3 triazine NH_2_ groups
3232	N-H symmetric stretching
1780	Stretching vibration of the carbonyl group C=O
1741	NH_2_ scissoring
1447	C-N symmetric stretching
918	Ring breathing type of vibration
808	Ring-sextant, out-of-plane bending type of vibration
766	Ring-sextant, out-of-plane bending type of vibration
594	Ring bending vibration
527	Side chain in plane C-N bending vibration

**Table 2:** Thermal characteristics of PA11 and PA1-FR.

Sample	Scan	Tg (°C)	Tm (°C)	ΔHm (J g^−1^)	Tc (°C)	ΔHc (J g^−1^)	Tg/Tm
**PA11**	First 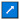	46	184/188	89.7	-	-	0.69
	Second 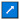	-	180/187	37.5	-	-	
	First 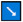	-	-	-	162	−42.4	
**PA11-FR**	First 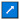	42	189	53.6	-	-	0.68
	Second 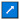	-	187	57.9	-	-	
	First 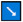	-	-	-	174	−43.6	

## Data Availability

The data presented in this study are available on request from the corresponding author.
